# Basal cell carcinoma of the prostate diagnosed incidentally with holmium laser enucleation of the prostate: How can we detect prior to benign prostatic hyperplasia surgery?

**DOI:** 10.1002/iju5.12282

**Published:** 2021-03-18

**Authors:** Eiichiro Ohara, Hiroshi Aoki, Yosuke Arakawa, Atsushi Kato, Rie Shibuya, Shigeto Ishidoya

**Affiliations:** ^1^ Department of Urology Sendai City Hospital Sendai Miyagi Japan; ^2^ Department of Pathology Sendai City Hospital Sendai Miyagi Japan

**Keywords:** basal cell carcinoma, Ki‐67, prostate

## Abstract

**Introduction:**

Basal cell carcinoma of the prostate is a rare prostate malignancy. Its diagnosis and treatment have not been well established yet.

**Case presentation:**

An 80‐year‐old man was referred to our hospital for undergoing holmium laser enucleation of the prostate with persistent lower urinary tract symptoms. Ultrasonography showed enlarged prostate (41.3 cc) with mid‐lobe hypertrophy. His prostate‐specific antigen and testosterone levels were 0.437 ng/mL and 873 ng/dL, respectively. Histological examination of the enucleated tissue confirmed basal cell carcinoma. The cells were positive for bcl‐2, 34βE12, p63, and cytokeratin 5/6. Ki‐67 labeling index was 5%. Subsequent staging computed tomography scan and bone scintigram showed neither lymph node nor distant metastasis. Currently, the patient is under close follow‐up with imaging, endoscopy, and urodynamic study.

**Conclusion:**

It is difficult for physicians to detect prostate basal cell carcinoma before benign prostatic hyperplasia surgery. In case of organ‐confined disease, age and Ki‐67 labeling index could be suggestive of subsequent treatment decision‐making.

**Abbreviations & Acronyms**
BCC: basal cell carcinoma
BPH: benign prostatic hyperplasia
HoLEP: holmium laser enucleation of the prostate
IPSS: International Prostate Symptom Score
OABSS: Overactive Bladder Symptom Score
PSA: prostate‐specific antigen
TURP: transurethral resection of the prostate

**Keynote message**

We report a case of prostate BCC which was incidentally found by HoLEP specimen. The clinical manifestation of the disease is almost identical with that of benign prostate mid‐lobe hypertrophy; hence, it is quite difficult to diagnose prior to TURP or HoLEP. The subsequent management of the disease is also controversial; however, age and Ki‐67 labeling index could be helpful in terms of treatment decision‐making if in case of organ‐confined disease.

## Case presentation

**Fig. 1:**
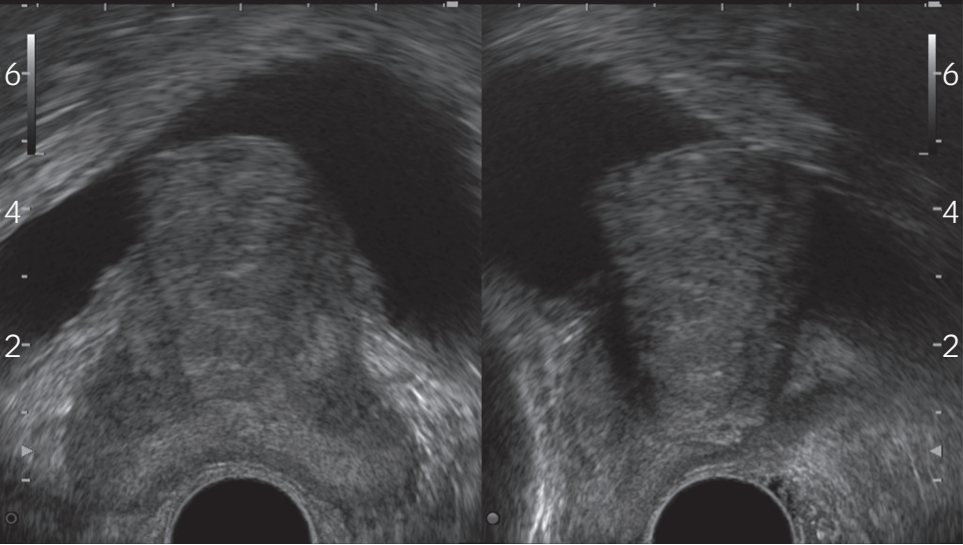
Prostate ultrasonography showed a marked mid‐lobe hypertrophy that extended toward bladder lumen.

An 80‐year‐old man was referred to our hospital for undergoing HoLEP with persistent lower urinary tract symptoms. He has been diagnosed with BPH and was prescribed tamsulocin (0.2 mg), dutasteride, and/or mirabegron for 2 years; however, no improvement was achieved with the medical treatment. At the initial visit, he also took several medications for diabetes mellitus, hypertension, and hyperuricemia. Ultrasonography showed enlarged prostate (41.3 cc) with evident mid‐lobe hypertrophy (Fig. 1). His IPSS, quality of life score, and OABSS were 5535555 = 35, 6 point, and 0350 = 8, respectively. Uroflowmetry showed poor urinary stream with 137 cc of residual urine. Digital rectal examination revealed an over‐walnut‐sized elastic firm prostate without any nodule. His prostate‐specific antigen and testosterone levels were 0.437 ng/mL and 873 ng/dL, respectively. He underwent HoLEP and 10 g of mid‐lobe‐prostate was removed.

**Fig. 2:**
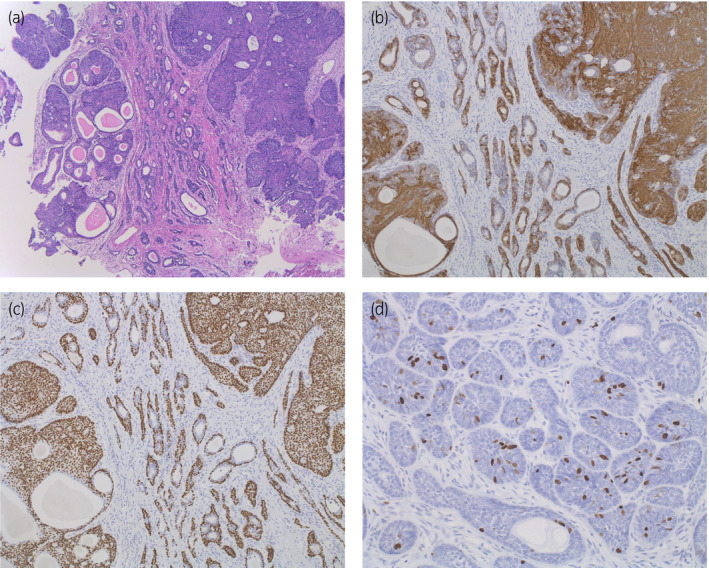
(a) Microscopic section of hematoxylin and eosin‐stained HoLEP specimen. Granular and cystic neoplastic cells were widely observed. (b) Immunohistochemical examination with 34βE12 demonstrated strong staining showing double‐layered structure of the neoplasm. (c) Immunohistochemical examination with p63 demonstrated positive staining also indicating basal‐cell origin. (d) Ki‐67 immunostaining index was 5% in the specimen.

**Fig. 3:**
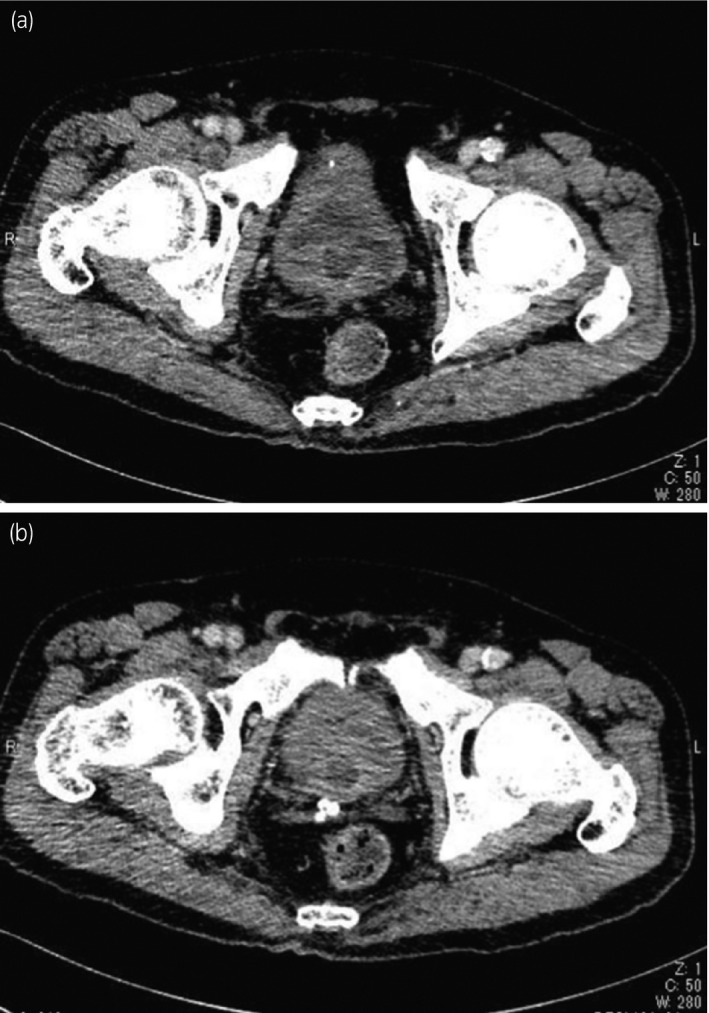
Post‐HoLEP computed tomography scan. Mid‐lobe of the prostate was well enucleated. (b) Sufficient lumen of the prostatic urethra was observed. Neither extra‐prostatic invasion lymph node metastases were detected.

Histological examination demonstrated a glandular structure and adenoid cystic‐like pattern, nearly 40% of which were consistent with BCC. The cells were positive for basal cell markers bcl‐2, 34βE12, p63, and cytokeratin 5/6, and negative for endothelial marker cytokeratin 20 immunohistochemically. Ki‐67 labeling index was 5% (Fig. 2). We could not find any concomitant adenocarcinoma or ductal carcinoma component. Subsequent staging computed tomography scan (Fig. 3) and bone scintigram showed neither lymph node nor distant metastasis. After 6 months of operation, his IPSS, quality of life score, and OABSS were 0205404 = 15, 6 point, and 0344 = 11, respectively, which were less favorable than we expected. Although he could void with 31 cc of residual urine, endoscopic findings showed a hard and stenotic prostatic urethra. Currently, after 1 year of HoLEP, the patient is under close follow‐up with imaging, endoscopy, and urodynamic study.

## Discussion

We encounter numerous number of patients with prostate adenocarcinoma in a daily practice; however, BCC of the prostate is a rare prostate malignancy.

Prostate BCC is associated with unique characters that were reported to date.^1^ It is derived from prostate basal cell of the transition zone of the prostate without PSA reactivity. Although several clinicopathological research data were reported, the oncological nature and treatment strategy have not been established yet.^2^

Initially, the present case was diagnosed as benign mid‐lobe hypertrophy without doubt, which we urologists commonly face, and then received HoLEP without magnetic resonance imaging and prostate biopsy. The current case showed almost identical manifestation compared with that of BPH. The authors are wondering how to detect the disease prior to BPH surgery. We admit that lacking in preoperative imaging studies was a limitation of our case study. Several previous reports demonstrated usefulness of imaging modalities like magnetic resonance imaging or fluorodeoxyglucose‐positron emission tomography.^3^, ^4^, ^5^ Considering the severe symptoms and the relatively small prostate, at least magnetic resonance imaging prior to HoLEP should have taken. The indication of imaging should be further clinically investigated.

The second question of BCC is that how intensive we should treat it. Recently, Hennes *et al*. published an informative case regarding BCC of the prostate with concurrent adenocarcinoma.^3^ In that, they performed robot‐assisted radical prostatectomy after diagnosis by the specimens of TURP. Tsuruta *et al.* also reported a case of 48‐year‐old man presenting with an 8 × 7 × 5 cm prostate tumor. He was treated with neoadjuvant chemotherapy for small‐cell carcinoma and with subsequent pelvic exenteration; however, he was finally diagnosed with BCC (Ki‐67:10%) and died of the disease.^6^ Ali and Epstein examined 29 cases of BCC clinicopathologically and mentioned that large solid nests with central necrosis and high Ki‐67 (≥20%) could be morphological characters of aggressive behavior.^7^ On the other hand, Dong *et al*. reported that a 62‐year‐old man with BCC showed multiple lung metastases 2 years after radical prostatectomy and adjuvant radiation.^8^ The current 80‐year‐old patient showed 5% of Ki‐67 labeling index without other prostate malignant component and evident distant metastases so that we undertake no adjuvant radical treatment but close follow‐up. Indeed no definitive threshold has been established so far. As the patient’s follow‐up was less than 14 months, we must see him as carefully as possible.

Prostate BCC is rare but is associated with unique characters that cause physicians to be embarrassed regarding diagnosis and treatment. Previous reports rather recommend radical surgery against the disease;^9^ however, in case of organ‐confined disease, age and Ki‐67 labeling index could be suggestive of subsequent decision‐making.

## Conflict of interest

The authors declare no conflict of interest.
